# Supplementary material for: Role and mechanism of NCAPD3 in promoting malignant behaviors in gastric cancer
Source: Front Pharmacol. 2024 Apr 22;15:1341039. doi: 10.3389/fphar.2024.1341039 (PMC11070777; doi:10.3389/fphar.2024.1341039)
Supplement: Supplementary file 11 [file DataSheet2.ZIP › GSEA/immunologic/my_analysis.Gsea.1599462003817/GSE2770_TGFB_AND_IL4_VS_IL4_TREATED_ACT_CD4_TCELL_6H_UP.html]

Details for gene set GSE2770\_TGFB\_AND\_IL4\_VS\_IL4\_TREATED\_ACT\_CD4\_TCELL\_6H\_UP[GSEA]

|  || Dataset | filtered\_dataset.sample\_info.cls#WT\_versus\_NCAPD3\_MUT |
| Phenotype | sample\_info.cls#WT\_versus\_NCAPD3\_MUT |
| Upregulated in class | WT |
| GeneSet | GSE2770\_TGFB\_AND\_IL4\_VS\_IL4\_TREATED\_ACT\_CD4\_TCELL\_6H\_UP |
| Enrichment Score (ES) | 0.46917868 |
| Normalized Enrichment Score (NES) | 2.1705892 |
| Nominal p-value | 0.0 |
| FDR q-value | 0.8400798 |
| FWER p-Value | 0.746 |
Table: GSEA Results Summary

  

Fig 1: Enrichment plot: GSE2770\_TGFB\_AND\_IL4\_VS\_IL4\_TREATED\_ACT\_CD4\_TCELL\_6H\_UP      
 Profile of the Running ES Score & Positions of GeneSet Members on the Rank Ordered List

  

| SYMBOL | TITLE | RANK IN GENE LIST | RANK METRIC SCORE | RUNNING ES | CORE ENRICHMENT || 1 | 51585 | PCF11 | 3 | 1.212 | 0.0768 | Yes |
| 2 | 63926 | ANKEF1 | 4 | 1.180 | 0.1537 | Yes |
| 3 | 64172 | OSGEPL1 | 6 | 1.152 | 0.2280 | Yes |
| 4 | 115209 | OMA1 | 45 | 0.932 | 0.2613 | Yes |
| 5 | 129607 | CMPK2 | 124 | 0.779 | 0.2557 | Yes |
| 6 | 7248 | TSC1 | 158 | 0.718 | 0.2787 | Yes |
| 7 | 11162 | NUDT6 | 162 | 0.716 | 0.3231 | Yes |
| 8 | 79664 | ICE2 | 217 | 0.662 | 0.3272 | Yes |
| 9 | 126792 | B3GALT6 | 223 | 0.658 | 0.3665 | Yes |
| 10 | 4281 | MID1 | 294 | 0.595 | 0.3547 | Yes |
| 11 | 10818 | FRS2 | 363 | 0.558 | 0.3421 | Yes |
| 12 | 8573 | CASK | 368 | 0.554 | 0.3753 | Yes |
| 13 | 10618 | TGOLN2 | 383 | 0.547 | 0.4008 | Yes |
| 14 | 60526 | LDAH | 403 | 0.535 | 0.4219 | Yes |
| 15 | 140775 | SMCR8 | 487 | 0.480 | 0.3933 | Yes |
| 16 | 140688 | NOL4L | 495 | 0.475 | 0.4192 | Yes |
| 17 | 162962 | ZNF836 | 540 | 0.452 | 0.4169 | Yes |
| 18 | 90806 | ANGEL2 | 583 | 0.434 | 0.4148 | Yes |
| 19 | 219743 | TYSND1 | 607 | 0.419 | 0.4255 | Yes |
| 20 | 10274 | STAG1 | 633 | 0.406 | 0.4339 | Yes |
| 21 | 10280 | SIGMAR1 | 655 | 0.399 | 0.4447 | Yes |
| 22 | 57496 | MKL2 | 658 | 0.398 | 0.4692 | Yes |
| 23 | 8835 | SOCS2 | 840 | 0.275 | 0.3565 | No |
| 24 | 81544 | GDPD5 | 961 | -0.381 | 0.2947 | No |
| 25 | 10616 | RBCK1 | 1042 | -0.438 | 0.2655 | No |
Table: GSEA details [plain text format]

  

Fig 2: GSE2770\_TGFB\_AND\_IL4\_VS\_IL4\_TREATED\_ACT\_CD4\_TCELL\_6H\_UP      
 Blue-Pink O' Gram in the Space of the Analyzed GeneSet

  

Fig 3: GSE2770\_TGFB\_AND\_IL4\_VS\_IL4\_TREATED\_ACT\_CD4\_TCELL\_6H\_UP: Random ES distribution      
 Gene set null distribution of ES for **GSE2770\_TGFB\_AND\_IL4\_VS\_IL4\_TREATED\_ACT\_CD4\_TCELL\_6H\_UP**
